# The Urgency of School-Based Interventions for Non-alcoholic Fatty Liver Disease: A Primary Prevention Approach

**DOI:** 10.34172/jrhs.8928

**Published:** 2025-06-10

**Authors:** Leyla Ahmadi Lari, Marzieh Asadilari, Fatemeh Saranjam

**Affiliations:** ^1^Department of Anesthesiology, School of Nursing, Larestan University of Medical Sciences, Larestan, Iran; ^2^Department of Nursing, School of Nursing, Larestan University of Medical Sciences, Larestan, Iran

## Dear Editor,

 Non-alcoholic fatty liver disease (NAFLD) has emerged as the most prevalent chronic liver disease globally.^1^ It is characterized by the accumulation of triglycerides in liver cells without a history of excessive alcohol consumption, drug use, or viral hepatitis.^2^ This condition is estimated to affect one-third of the world’s population.^1^ From 2000 to 2021, the prevalence of NAFLD among children and adolescents in North America was 8.35% and 7.01% in Asia, respectively. Notably, it is significantly more prevalent among obese individuals, with a prevalence of 52.49%.^3^ A study conducted in Iran reported that the overall prevalence of NAFLD was 6.7% in children and 42% in obese children.^4^

 Evidence suggests that NAFLD is linked to cardiovascular and metabolic complications such as type 2 diabetes, dyslipidemia, and hypertension. The severity of these conditions is directly correlated with the degree of obesity. Given the absence of proven pharmacological treatments for pediatric NAFLD, lifestyle modifications, including dietary and exercise interventions, remain the cornerstone of management.^5^ Lifestyle interventions, with a focus on healthy eating habits and increased physical activity, can lead to weight loss and reduced hepatic fat deposition in children. Exercise also plays a pivotal role in preventing childhood obesity, improving insulin sensitivity, and reducing NAFLD risk factors.^6^ Additionally, consuming adequate fruits and vegetables while reducing the frequency of sugary drinks, desserts, and fried foods can significantly decrease the risk of developing hepatic steatosis.^7^

## Recommendations to researchers and school health professionals

 Given that adolescents are increasingly spending their time on sedentary activities such as using mobile phones, tablets, and playing video games, they are consequently engaging in less physical activity and not prioritizing their health. Therefore, we suggest that societal policies promote programs aimed at playing outdoor games, walking, and cycling in schools. Additionally, healthcare professionals should identify and address barriers to physical activity. Regular BMI measurements and targeted weight loss goals for overweight students are essential. We recommend that school health professionals conduct educational sessions for parents, encouraging them to increase their consumption of fruits and vegetables while reducing their intake of fast food, fried foods, and high-fat foods at home. School cafeterias can also contribute to the development of unhealthy eating habits, such as the consumption of high-calorie and unhealthy foods, leading to NAFLD. Therefore, health professionals play a crucial role in monitoring school cafeterias. They must ensure that the food products available in cafeterias comply with nutritional and hygiene standards and also organize workshops and training sessions to promote healthy diets for students and especially their parents, as parents can serve as positive role models for their children by demonstrating healthy behaviors and habits. By implementing various interventions for children and adolescents, particularly in schools, and by adopting a healthy lifestyle with an emphasis on weight management and increased physical activity, we hope to prevent chronic diseases, especially NAFLD.

## Competing Interests

 There is no conflict of interests.

## Ethical Approval

 Not applicable.

## Funding

 No funding was received for this article.
